# Factors Associated With Type 2 Diabetes in Older Japanese With Similar Genetic Risk Scores: The Bunkyo Health Study

**DOI:** 10.1210/jendso/bvaf019

**Published:** 2025-01-28

**Authors:** Thu Hien Bui, Hideyoshi Kaga, Saori Kakehi, Yuki Someya, Hiroki Tabata, Yasuyo Yoshizawa, Hitoshi Naito, Tsubasa Tajima, Naoaki Ito, Satoshi Kadowaki, Yuya Nishida, Ryuzo Kawamori, Hirotaka Watada, Yoshifumi Tamura

**Affiliations:** Department of Metabolism and Endocrinology, Sportology Center, Graduate School of Medicine, Juntendo University, Tokyo 113-8421, Japan; Department of Metabolism and Endocrinology, Sportology Center, Graduate School of Medicine, Juntendo University, Tokyo 113-8421, Japan; Sports Medicine and Sportology, Sportology Center, Graduate School of Medicine, Juntendo University, Tokyo 113-8421, Japan; Sportology Center, Graduate School of Medicine, Juntendo University, Tokyo 113-8421, Japan; Sportology Center, Graduate School of Medicine, Juntendo University, Tokyo 113-8421, Japan; Sportology Center, Graduate School of Medicine, Juntendo University, Tokyo 113-8421, Japan; Juntendo Advanced Research Institute for Health Science, Tokyo 113-8421, Japan; Juntendo Advanced Research Institute for Health Science, Tokyo 113-8421, Japan; Faculty of International Liberal Arts, Juntendo University, Tokyo 113-8421, Japan; Department of Metabolism and Endocrinology, Sportology Center, Graduate School of Medicine, Juntendo University, Tokyo 113-8421, Japan; Department of Metabolism and Endocrinology, Sportology Center, Graduate School of Medicine, Juntendo University, Tokyo 113-8421, Japan; Department of Metabolism and Endocrinology, Sportology Center, Graduate School of Medicine, Juntendo University, Tokyo 113-8421, Japan; Department of Metabolism and Endocrinology, Sportology Center, Graduate School of Medicine, Juntendo University, Tokyo 113-8421, Japan; Department of Metabolism and Endocrinology, Sportology Center, Graduate School of Medicine, Juntendo University, Tokyo 113-8421, Japan; Department of Metabolism and Endocrinology, Sportology Center, Graduate School of Medicine, Juntendo University, Tokyo 113-8421, Japan; Sports Medicine and Sportology, Sportology Center, Graduate School of Medicine, Juntendo University, Tokyo 113-8421, Japan; Sportology Center, Graduate School of Medicine, Juntendo University, Tokyo 113-8421, Japan; Department of Metabolism and Endocrinology, Sportology Center, Graduate School of Medicine, Juntendo University, Tokyo 113-8421, Japan; Sports Medicine and Sportology, Sportology Center, Graduate School of Medicine, Juntendo University, Tokyo 113-8421, Japan; Sportology Center, Graduate School of Medicine, Juntendo University, Tokyo 113-8421, Japan; Department of Metabolism and Endocrinology, Sportology Center, Graduate School of Medicine, Juntendo University, Tokyo 113-8421, Japan; Sports Medicine and Sportology, Sportology Center, Graduate School of Medicine, Juntendo University, Tokyo 113-8421, Japan; Sportology Center, Graduate School of Medicine, Juntendo University, Tokyo 113-8421, Japan; Juntendo Advanced Research Institute for Health Science, Tokyo 113-8421, Japan; Faculty of International Liberal Arts, Juntendo University, Tokyo 113-8421, Japan

**Keywords:** genetic risk score, type 2 diabetes, visceral fat area

## Abstract

**Context:**

Genome-wide association studies have identified numerous single-nucleotide variations (SNVs, formerly single-nucleotide polymorphisms) linked to type 2 diabetes (T2D), thus improving the accuracy of genetic risk scores (GRS) in predicting T2D.

**Objective:**

This study aimed to investigate the association between the novel GRS and the prevalence of T2D and clarify the characteristics that differentiate individuals with and without T2D with similar genetic risk.

**Methods:**

This cross-sectional study analyzed 1610 Japanese individuals aged 65 to 84 years. GRS were calculated using 110 SNVs associated with T2D in Japanese, and GRS classified individuals as having low, average, or high risk for T2D. The characteristics of participants with or without diabetes were compared by sex at each risk level.

**Results:**

The prevalences of T2D were 7.8%, 14.7%, and 16.7% at low-, average-, and high-risk levels, respectively. The odds ratios at the high- and average-risk levels were significantly higher than those at the low-risk level, even after adjusting for confounding factors. The diabetes group had a higher visceral fat area (VFA) and Homeostasis Model Assessment of Insulin Resistance (HOMA-IR) value, but a lower insulinogenic index, than the nondiabetes group across all risk levels. In the nondiabetes group, the II decreased significantly as GRS increased, but the HOMA-IR and Matsuda index values showed no association. In men with diabetes, VFA tended to decrease with higher GRS.

**Conclusion:**

A higher GRS was significantly associated with increased T2D prevalence in older Japanese individuals. Our data demonstrated that the contribution of VFA to the development of diabetes varies with genetic risk.

Diabetes has become a global pandemic, affecting 537 million people worldwide, and this number is predicted to continue rising in the future. More than 90% of affected individuals have type 2 diabetes (T2D) [1]. In 2019, the prevalences of T2D were 13.8% and 7.7% in Japanese men and women, respectively, without any statistically significant changes over the previous 10 years [2]. The high prevalence of this disease imposes a major burden not only on health care expenditures but also on individuals, who often face a lifelong struggle with debilitating complications that affect quality of life. Therefore, achieving early prevention by identifying high-risk populations and enhancing individual risk assessment is essential not only for improving health care systems but also for reducing the human costs associated with this disease.

The advent of genome-wide association studies (GWASs) has revolutionized our capacity to evaluate disease susceptibility [3]. To date, studies have explored around 500 loci associated with T2D, and these have enabled improvements in individual T2D prediction through the use of genetic risk scores (GRSs) [4]. However, the effectiveness of using genetic risk factors to predict diabetes remains controversial, as this process depends on race and the number of single-nucleotide variations (SNVs; formerly single-nucleotide polymorphisms) that are used. Recent research by Suzuki et al [5] identified novel SNVs associated with T2D susceptibility within the Japanese population; however, the application of these findings to GRS-based assessments of diabetes prevalence remains unexplored. There is a clear need for research evaluating how these newly discovered SNVs can enhance the accuracy of GRS in predicting diabetes. Furthermore, the availability of genetic data has facilitated personalized risk assessments for diabetes, fueling interest in tailored interventions such as lifestyle adjustments and targeted pharmacological therapies, particularly for high-risk individuals. Nevertheless, disparities persist: Certain genetically predisposed populations remain diabetes free, while others with seemingly low genetic susceptibility develop the disease [6, 7]. Therefore, it is not solely the GRS; other conventional factors associated with T2D must also be considered to explain these discrepancies. Understanding the factors contributing to these discrepancies could help identify lifestyle modifications that mitigate diabetes onset in genetically vulnerable individuals. It could also reveal the environmental triggers that put genetically resilient individuals at risk. These possibilities hold promise for advancing customized strategies in diabetes prevention.

Against this background, this study aimed to investigate the association between the novel GRS and the prevalence of T2D among older Japanese individuals, as well as to clarify the characteristics that differentiate individuals with and without T2D who have similar genetic risk. Using data from the Bunkyo Health Study (BHS), we stratified a cohort of 1610 individuals aged 65 to 84 years into 3 genetic risk categories based on their GRS, which were calculated from 110 SNVs associated with T2D. This approach could potentially enable the development of tailored preventive strategies for T2D based on varying levels of genetic risk.

## Materials and Methods

### Outline of the Bunkyo Health Study

The BHS is a cohort study started in 2015 to investigate the correlations between muscle mass, muscle strength, insulin sensitivity, and the incidence of diseases associated with the need for long-term care. The BHS included 1629 community-dwelling older adults aged 65 to 84 years who lived in Bunkyo-ku, Tokyo, Japan. The participants were recruited by mail. After providing informed consent, they underwent brain magnetic resonance imaging, blood and urine analysis, and a series of examinations to assess muscle mass and strength, glucose tolerance, cognitive function, lifestyle and physical activity levels, and physical fitness and function. The exclusion criteria were pacemaker, defibrillator placement, and diabetes requiring insulin therapy. The protocol for the BHS has been published elsewhere [8]. Nineteen people who did not have genetic information were excluded, leading to the final analysis of 1610 participants. The study protocol was approved by the ethics committee of Juntendo University in September 2015 (first approval No. 2015061 and the latest revised version No. M15-0057-M09). This research was conducted according to the principles outlined in the Declaration of Helsinki. All participants provided written informed consent and were notified that they had the right to withdraw from the study at any time.

### Genotyping and Calculation of Genetic Risk Score for Type 2 Diabetes

Genomic DNA was extracted from peripheral blood cells using a DNA extraction kit (DNeasy Blood and Tissue Kit; Qiagen). Genotypes were determined using the Illumina Infinium Asian Screening Array-24 v1.0 BeadChip (Illumina), and were called using GenomeStudio (version 2013; Illumina). Sample quality control included missing rates (>0.05), unusually high fractions of heterozygous variant calls, and sex chromosome aneuploidy. Genotyped SNVs on chrY, chrXY, and chrMT (mitochondrial chromosomes), as well as insertions and deletions, were excluded manually. The autosomal SNVs (351, 928) that passed quality control criteria were imputed using BEAGLE v5.1 software, with the 1000 Genomes Phase 3 v5a data sets of the Haplotype Reference Consortium serving as reference panels. The final number of variants inferred by imputation was 32 086 350. To calculate the GRS, we first selected a set of SNVs that had been previously associated with T2D [5]. These SNVs were selected based on their significance in GWASs. However, some SNVs, particularly in UBE2E2, HNF1A, MNX1, SLC30A8, and TIMM17B/PCSK1N loci, were not included in the variants analyzed in this study due to their absence in the reference panel. Consequently, 110 of the 115 SNVs were selected to determine GRS in this study. The equation for GRS calculation was as follows [9]:


$$GRS=\overset{k}{\underset{i=1}{\sum }}\;\beta_{i}N_{i}$$

where k is the number of independent genetic variants, in this case k = 110 SNV, that strongly predict diabetes risk, β*i* is the effect size of each SNV calculated from a previous Japanese GWAS [5]. *Ni* is the number of risk alleles in each SNV. The GRS in the present study ranged from 100.0 to 144.5. A higher GRS indicates a higher genetic risk for T2D.

### Type 2 Diabetes Diagnoses

Blood samples were collected after overnight fasting, followed by a standard 75-g oral glucose tolerance test (OGTT) to measure plasma glucose and serum insulin levels. Glycated hemoglobin A_1c_ (HbA_1c_) levels were measured the same day. According to the diabetes diagnostic criteria of the Japan Diabetes Society, T2D was defined as follows: fasting plasma glucose (FPG) greater than or equal to 126 mg/dL and/or a 2-hour glucose level after the 75-g OGTT greater than or equal to 200 mg/dL, and HbA_1c_ greater than or equal to 6.5%, or use of oral hypoglycemic agents [10].

### Assessment of Metabolic Characteristics

Participants' blood samples were collected, and a thorough evaluation of metabolic parameters was performed to investigate the relationship between metabolic characteristics and genetic predispositions in T2D. Fasting blood glucose levels and the glucose area under the curve (AUC) during a 75-g OGTT were analyzed to evaluate overall glucose homeostasis, while HbA_1c_ levels were measured for long-term glucose monitoring. Insulin dynamics were assessed by measuring fasting insulin levels, fasting C-peptide immunoreactivity (CPR), insulin-AUC, CPR-AUC, and insulin clearance. In addition, insulin resistance and insulin secretion were evaluated using the HOMA-IR, Matsuda index, and insulinogenic index (II). The HOMA-IR was calculated as fasting serum insulin (µU/mL) × FPG (mg/dL)/405. The Matsuda index was calculated using the following equation: 10 000/square root of (FPG (mg/dL) × fasting insulin (µU/mL)) × (mean glucose (mg/dL) × mean insulin during OGTT (µU/mL)) [11]. The II, reflecting early-phase glucose-dependent insulin secretion, was calculated using the following equation: (change in insulin/change in glucose from 0 to 30 minutes) [12].

Lipid metabolism was assessed by measuring fasting free fatty acids (FFA), FFA-AUC, and adipose tissue insulin resistance (Adipo-IR), and a comprehensive lipid profile included total cholesterol, high-density lipoprotein cholesterol (HDL-C), low-density lipoprotein cholesterol (LDL-C), and triglycerides.

### Abdominal Fat Area

Visceral and subcutaneous fat areas were measured by specialized image analysis software (AZE Virtual Place, AZE) after an intra-abdominal magnetic resonance imaging scan as described previously [8].

### Demographic Information

Age, body mass index (BMI), smoking status, history of hypertension, and history of dyslipidemia were determined using a questionnaire. The percentage of body fat (PBF) was measured by bioelectrical impedance analysis (InBody770, InBody Japan Inc). The International Physical Activity Questionnaire was used to assess physical activity level (metabolic equivalents [METs]·hour/week) [13]. Nutritional intake was assessed by the brief-type self-administered diet history questionnaire containing 58 food items [14].

### Statistical Analysis

The data was presented as median (quartiles) due to the skewed distribution of most variables. Participants of each sex were divided into 3 groups by GRS tertiles (low-risk, average-risk, and high-risk levels). Sex-stratified analysis was performed due to the inherent differences in the prevalence of diabetes, PBF, and visceral fat area between men and women. Differences in sociodemographic characteristics between the 3 groups with nonnormally distributed data were tested using the Kruskal-Wallis test, and differences in proportions were assessed using the chi-square test. Logistic regression was used to estimate odds ratios (ORs) and 95% CIs for the association between GRS and the prevalence of diabetes, with adjustments for potential confounders. In this study, 2 models were generated using a regression analysis. Model 1 was adjusted for age (at baseline) and BMI. Model 2 was adjusted for age, BMI, PBF, subcutaneous fat area, visceral fat area (VFA), and physical activity. We determined the potential confounders based on prior knowledge of factors related to the development of T2D, which is the most commonly used method in epidemiology. To assess the differences in body composition, energy intake, physical activity and biochemical characteristics between participants with or without diabetes (DM and non-DM groups, respectively) who had the same level of genetic risk for diabetes, the *t* test and Mann-Whitney *U* test were used for normally and nonnormally distributed variables, respectively. The Jonckheere-Terpstra test was applied to assess the change in each characteristic when the level of GRS increased in the non-DM and DM groups. Analysis was performed using SPSS version 29.0 (IBM). *P* less than .05 was considered statistically significant.

## Results

### Population Characteristics

**Figure 1.**
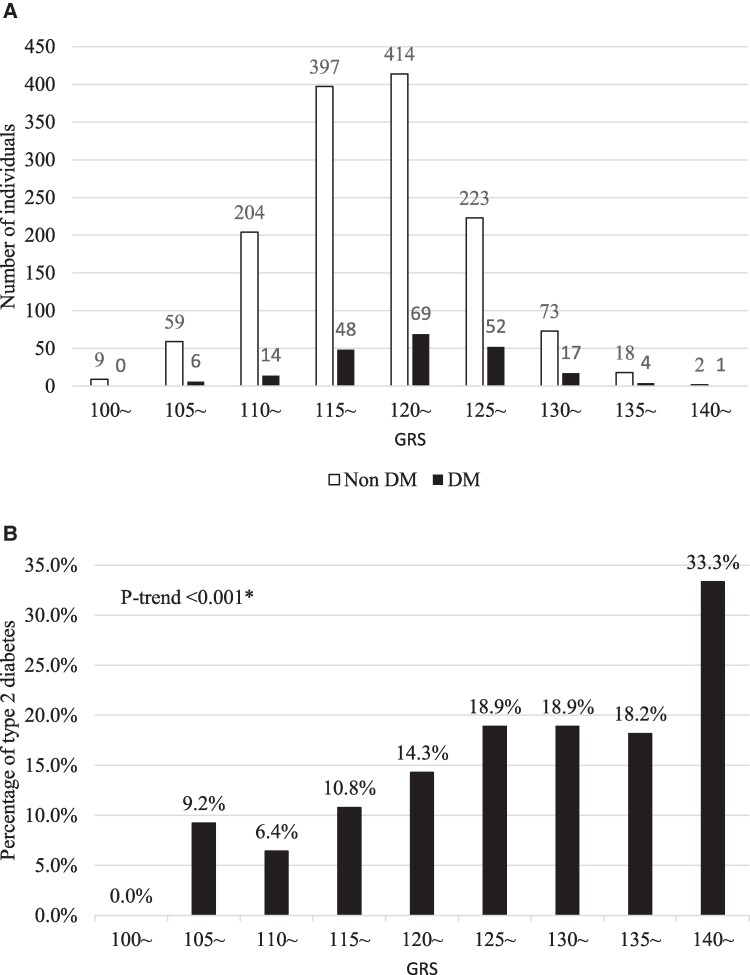
(A) Distribution of individuals according to genetic risk score (GRS) in nondiabetes (non-DM) (white bars) and DM groups (black bars), categorized by 5-point increments. (B) Prevalence of type 2 diabetes according to GRS. *P for trend was calculated by logistic regression.

The study population had a baseline average age of 73.1 ± 5.4 years and 57.8% of the participants were women. The overall prevalence of diabetes was 13.1% (men: 18.6%, women: 9.1%). Figure 1A shows the distribution of individuals with and without diabetes according to the GRS, categorized in 5-point increments. The average GRS was 120.6 ± 6.4, with a normal distribution. In addition, the average GRS of the DM group (122.6 ± 6.1) was statistically significantly higher than that of the non-DM group (120.4 ± 6.4) (*P* < .001). The prevalence of T2D significantly increased with higher levels of the GRS (*P* for trend <.001) (Fig. 1B).

To further elucidate the characteristics of individuals with and without T2D at similar GRS levels, we performed an analysis after dividing the study population into tertiles based on GRS. The characteristics of the study participants by GRS level are shown in Table 1. The age and sex ratio were similar for each of the 3 risk levels. Regarding demographic characteristics, there were no differences in BMI, lifestyle, or the percentages of participants with hyperlipidemia, but individuals at the high-risk level were significantly less likely to have hypertension than those at other levels.

**Table 1.** General baseline characteristics of participants by genetic risk score level in the Bunkyo health study (n = 1610)

	Low risk	Average risk	High risk	*P^a^*
	n = 536	n = 538	n = 536	
Female, %	310 (57.8)	310 (57.6)	311 (58.0)	.991
Age, y	73.0 (68.0-78.0)	73.0 (68.0-77.0)	73.0 (68.0-78.0)	.744
Height, cm	157.3 (151.8-165.1)	157.5 (151.5-164.6)	157.0 (151.8-164.5)	.853
Weight, kg	56.8 (50.6-65.4)	56.1 (48.9-64.1)	55.8 (49.4-63.7)	.197
Body mass index	22.8 (21.0-25.0)	22.5 (20.7-24.3)	22.5 (20.6-24.8)	.085
Body fat, %	29.1 (23.4-33.9)	27.7 (23.2-32.9)	28.1 (23.1-33.5)	.351
Subcutaneous fat area, cm^2^	148.1 (112.1-197.2)	136.6 (106.1-179.7)	140.6 (102.8-182.5)	.004
Visceral fat area, cm^2^	74.7 (51.6-102.4)	71.8 (48.9-96.3)	72.2 (51.7-99.5)	.133
History of smoking, %	221 (41.2)	224 (41.6)	215 (40.1)	.871
Current smoking, %	45 (8.4)	35 (6.5)	40 (7.5)	.494
Living alone, %	115 (21.5)	120 (22.3)	102 (19.0)	.392
Diabetes, %	7.8%	14.7%	16.7%	<.001
Hypertension, %	364 (67.9)	368 (68.4)	331 (61.8)	.038
Physical activity, METs.h/wk	29.7 (16.5-51.1)	30.1 (16.5-51.7)	29.7 (17.6-56.9)	.700
Hyperlipidemia, %	333 (62.1)	338 (62.8)	340 (63.4)	.907
Energy intake, kcal/d	1904.8 (1580.0-2336.9)	1856.7 (1519.9-2257.6)	1902.2 (1536.9-2285.8)	.238

Data are presented as median (quartiles) and count (percentage).

Abbreviation: METs, metabolic equivalents.

^*a*^ *P* values were calculated by the chi-square test and Kruskal-Wallis test for categorical and continuous variables, respectively.

### Odds Ratios for Type 2 Diabetes by Genetic Risk Score Levels With Covariate Adjustment

The prevalences of T2D were 7.8%, 14.7%, and 16.7% at low-, average-, and high-risk levels, respectively. In men and women, the ORs for T2D of individuals at average risk and high-risk levels were 2 to 3 times significantly higher than that at the low-risk level (crude model, Table 2). The ORs at the high-risk level (OR = 2.530; 95% CI, 1.685-3.799) and average risk level (OR = 2.224; 95% CI, 1.472-3.362) were significantly higher than that at the low-risk level after adjusting for age, BMI, body fat, and physical activity levels (model 2, see Table 2).

**Table 2.** Unadjusted and adjusted odd ratios for type 2 diabetes by genetic risk score level

	Low risk	Average risk	High risk
Total	n = 536	n = 538	n = 536
Crude	Reference	2.024 (1.363-3.006)	2.358 (1.600-3.475)
Model 1	Reference	2.123 (1.423-3.167)	2.407 (1.624-3.569)
Model 2	Reference	2.224 (1.472-3.362)	2.530 (1.685-3.799)
**Male**	n = 226	n = 228	n = 225
Crude	Reference	1.764 (1.065-2.923)	1.930 (1.170-3.184)
Model 1	Reference	1.786 (1.074-2.972)	1.948 (1.171-3.240)
Model 2	Reference	1.977 (1.169-3.342)	2.162 (1.276-3.661)
**Female**	n = 310	n = 310	n = 311
Crude	Reference	2.630 (1.352-5.114)	3.347 (1.753-6.393)
Model 1	Reference	2.810 (1.436-5.498)	3.428 (1.785-6.581)
Model 2	Reference	2.682 (1.358-5.295)	3.221 (1.665-6.233)

Type 2 diabetes was diagnosed using the following criteria: fasting plasma glucose greater than or equal to 126 mg/dL and/or a 2-hour glucose level after a 75-g oral glucose tolerance test greater than or equal to 200 mg/dL, and glycated hemoglobin A_1c_ greater than or equal to 6.5%, or use of oral hypoglycemic agents. Odds ratios (95% CI) were obtained by performing multiple logistic regression with the low genetic risk score level as a reference; model 1 was adjusted for age (at baseline) and body mass index; model 2 was adjusted for age, body mass index, percentage body fat, subcutaneous fat area, visceral fat area, and physical activity (metabolic equivalents.hour/week).

### Differences Between Nondiabetes Mellitus and Diabetes Mellitus Groups at Each Risk Level

Body composition parameters were compared between the non-DM and DM groups. In men, the DM group had a significantly higher BMI at the average risk level and a higher PBF at low and average risk levels than the non-DM group. In women, the DM group had a significantly higher PBF at the average risk level compared with the non-DM group. The two groups showed no difference in BMI at any risk level. On the other hand, in both sexes, the DM group had a significantly higher VFA than the non-DM group at all GRS levels (Table 3).

**Table 3.** Comparison of body composition, energy intake, and physical activity characteristics in nondiabetes mellitus and diabetes groups by genetic risk score level (n = 1610)

	Low risk		Average risk		High risk		Among risk groups (*P* trend)*^a^*	
	Non-DM	DM	Non-DM	DM	Non-DM	DM	Non-DM	DM
**Male**								
n	197	29	181	47	175	50		
Age, y	73.0 (69.0-78.0)	71.0 (68.5-77.0)	72.0 (68.0-77.0)	75.0 (69.0-79.0)	72.0 (68.0-77.0)	73.5 (69.0-77.3)	.332	.375
Body mass index	23.0 (21.6-25.3)	24.3 (22.2-26.4)	23.3 (21.8-24.8)	24.2 (22.4-26.1)*^b^*	23.0 (21.2-24.9)	23.6 (21.8-25.1)	.386	.217
Body fat, %	24.1 (20.5-28.8)	26.5 (24.2-30.7)*^b^*	24.6 (21.0-27.8)	26.6 (22.6-31.6)*^b^*	23.8 (18.6-27.6)	25.8 (21.0-29.8)	.192	.172
Subcutaneous fat area, cm^2^	124.0 (97.7-162.1)	131.4 (97.8-169.5)	120.0 (99.4-144.4)	129.5 (97.7-171.1)	118.4 (89.1-149.8)	114.2 (94.2-151.6)	.074	.107
Visceral fat area, cm^2^	85.1 (63.7-108.0)	129.8 (88.2-150.9)*^b^*	80.7 (65.0-100.6)	107.7 (77.6-132.8)*^b^*	77 (59.6-101.4)	96.9 (61.8-124.6)*^b^*	.053	.012
Physical activity, METs.h/wk	32.5 (17.0-59.8)	24.8 (16.0-44.7)	33.9 (17.9-55.3)	31.1 (13.8-49.0)	33.0 (19.8-59.0)	37.3 (20.3-67.4)	.809	.107
Energy intake, kcal/d	2104.7 (1773.5-2611.1)	2052.6 (1559.8-2284.9)	2075.6 (1696.2-2508.9)	1949.7 (1685.2-2356.9)	2086.9 (1640.7-2511.2)	1924.3 (1587.5-2524.0)	.456	.933
**Female**								
n	297	13	278	32	271	40		
Age, y	73.0 (68.0-78.0)	74.0 (69.0-79.5)	72.0 (68.0-76.0)*^b^*	76.0 (72.3-80.0)*^b^*	73.0 (68.0-78.0)	75.5 (70.0-78.0)	.521	.894
Body mass index	22.4 (20.4-24.6)	23.2 (20.7-27.5)	21.6 (19.6-23.5)	22.5 (20.8-24.2)	22.1 (20.0-24.2)	22.9 (20.4-26.0)	.119	.917
Body fat, %	32.2 (27.5-35.9)	34.6 (28.2-38.4)	30.1 (24.8-34.2)*^b^*	33.7 (29.0-38.5)*^b^*	31.3 (26.1-35.1)	33.8 (27.8-39.8)	.135	.927
Subcutaneous fat area, cm^2^	170.6 (126.4-218.6)	204.5 (110.5-240.1)	150.0 (115.5-194.9)	159.6 (117.8-219.6)	152.4 (121.6-208.0)	167.4 (99.8-235.0)	.039	.460
Visceral fat area, cm^2^	64.4 (43.5-87.1)	91.4 (75.2-106.0)*^b^*	58.4 (39.9-82.4)	82.2 (63.8-110.4)*^b^*	63.6 (45.3-86.2)	83.8 (63.5-119.4)*^b^*	.878	.784
Physical activity, METs.h/wk	29.7 (15.5-49.3)	19.8 (17.8-48.4)	29.0 (16.4-51.8)	25.6 (15.1-39.6)	29.2 (16.5-51.9)	24.3 (13.5-56.9)	.527	.881
Energy intake, kcal/d	1801.1 (1479.7-2185.1)	1688.3 (1493.7-2014.2)	1756.8 (1448.7-2119.1)	1632.3 (1420.1-1938.5)	1814.5 (1484.0-2146.5)	1750.4 (1371.5-2188.8)	.747	.460

Data are presented as median (quartiles); the *t* test and Mann-Whitney *U* test were performed to compare non-DM and DM groups within the same genetic risk score level.

Abbreviations: DM, diabetes mellitus; METs, metabolic equivalents.

^*a*^The Jonckheere-Terpstra test was performed in the non-DM and DM groups to calculate *P* trends.

^*b*^ *P* less than .05 vs non-DM at the same genetic risk score level.

Regarding glucose metabolism-related indices, among participants of all GRS levels and sexes, the DM group had significantly higher HbA_1c_, fasting insulin, CPR, and HOMA-IR values, but lower Matsuda index and insulinogenic index values. Furthermore, the DM group had significantly higher FFA-AUC and Adipo-IR values. In terms of lipid metabolism in men, HDL-C in the DM group was significantly lower than that in the non-DM group at low- and average-risk levels (Table 4). In women, HDL-C was lower in the DM group than in the non-DM group only at the high-risk level, while the DM group had significantly higher triglycerides at both average- and high-risk levels (Table 5).

**Table 4.** Comparison of biochemical characteristics in the nondiabetes mellitus and diabetes mellitus groups by genetic risk score level in male participants (n = 679)

	Low risk		Average risk		High risk		*P* trend*^a^*	
	Non-DM	DM	Non-DM	DM	Non-DM	DM	Non-DM	DM
	n = 197	n = 29	n = 181	n = 47	n = 175	n = 50		
Glucose, mg/dL	98.0 (93.0-103.0)	133.0 (106.5-142.5)*^b^*	96.0 (92.0-103.0)	132.0 (111.0-149.0)*^b^*	96.0 (90.0-103.8)	130.0 (115.5-148.5)*^b^*	.141	.467
Glucose-AUC, mg/dL × 10^−4^	1.8 (1.6-2.1)	2.9 (2.3-3.3)*^b^*	1.8 (1.6-2.1)	2.9 (2.5-3.2)*^b^*	1.9 (1.6-2.2)	2.9 (2.5-3.3)*^b^*	.213	.611
HbA_1c_, %	5.6 (5.4-5.9)	6.8 (6.5-7.4)*^b^*	5.6 (5.4-5.9)	6.8 (6.4-7.4)*^b^*	5.6 (5.4-5.8)	6.8 (6.5-7.1)*^b^*	.934	.689
Fasting insulin, μU/mL	4.2 (2.7-6.2)	5.9 (4.2-7.5)*^b^*	4.2 (2.6-5.8)	5.3 (3.6-7.6)*^b^*	3.8 (2.8-5.4)	4.4 (3.0-7.2)	.149	.087
Insulin-AUC, μU/mL × 10^−3^	4.9 (3.2-6.9)	2.8 (2.3-6.0)*^b^*	4.5 (3.3-7.1)	3.1 (2.2-5.2)*^b^*	4.2 (3.2-6.8)	2.8 (1.8-4.2)*^b^*	.201	.184
HOMA-IR	1.0 (0.6-1.5)	1.8 (1.3-2.4)*^b^*	1.0 (0.6-1.4)	1.6 (1.1-2.6)*^b^*	0.9 (0.6-1.4)	1.6 (0.9-2.2)*^b^*	.121	.208
Matsuda index	6.3 (4.7-8.8)	4.8 (3.1-7.2)*^b^*	7.0 (4.4-10.0)	5.0 (3.4-7.4)*^b^*	7.2 (4.9-9.9)	5.6 (3.4-8.5)*^b^*	.156	.259
Insulinogenic index	0.6 (0.3-1.0)	0.2 (0.1-0.2)*^b^*	0.5 (0.3-1.0)	0.2 (0.1-0.3)*^b^*	0.4 (0.3-0.8)	0.1 (0.1-0.2)*^b^*	.001	.190
Fasting C-peptide, ng/mL	1.5 (1.1-1.9)	2.0 (1.5-2.3)*^b^*	1.4 (1.1-1.8)	1.9 (1.5-2.4)*^b^*	1.3 (1.0-1.7)	1.5 (1.1-2.0)	.027	.008
AUC-C-peptide, ng/mL × 10^−2^	7.8 (6.1-9.4)	6.1 (4.7-8.3)*^b^*	7.6 (6.2-10.0)	6.0 (5.1-7.9)*^b^*	7.4(6.0-9.3)	5.2 (3.8-7.0)*^b^*	.287	.050
Fasting FFA, µmol/L	473.0 (369.9-617.5)	572.7 (393.4-713.0)	434.7 (349.1-564.7)	511.0 (441.0-608.0)*^b^*	450.0 (353.3-580.0)	546.0 (444.5-722.4)*^b^*	.169	.557
FFA-AUC, µmol/L × 10^−4^	2.9 (2.3-3.5)	4.1 (3.5-5.1)*^b^*	2.7 (2.1-3.5)	4.4 (3.7-5.1)*^b^*	2.8 (2.2-3.6)	4.2 (3.1-5.2)*^b^*	.103	.928
Adipo-IR	2.0 (1.2-3.3)	3.3 (2.2-3.9)*^b^*	1.8 (1.0-2.7)	2.5 (1.6-4.8)*^b^*	1.6 (1.0-2.6)	2.7 (1.3-3.9)*^b^*	.020	.253
Total cholesterol, mg/dL	192.0 (169.0-214.5)	168.0 (152.0-208.5)*^b^*	194.0 (175.0-222.0)	171.0 (156.0-189.0)*^b^*	194.0 (171.0-217.5)	185.5 (165.8-207.3)	.630	.065
HDL-C, mg/dL	57.0 (48.0-67.0)	51.0 (41.0- 59.5)*^b^*	57.0 (48.0-68.0)	54.0 (41.0-62.0)*^b^*	58.5 (51.0-68.8)	54.5 (48.5-63.3)	.329	.090
LDL-C, mg/dL	113.0 (93.0-131.0)	103.0 (84.0-129.5)	113.0 (93.0-135.5)	98.0 (83.0-118.0)*^b^*	115.0 (93.3-138.8)	108.0 (91.0- 125.5)	.616	.272
Triglycerides, mg/dL	91.0 (71.0-126.0)	105.0 (81.0-130.0)	85.0 (62.5-120.0)	99.0 (75.0-128.0)	81.5 (60.3-121.0)	92.5 (75.3-119.0)	.029	.183

Data are presented as median (quartiles); the *t* test and Mann-Whitney *U* test were performed to compare non-DM and DM groups within the same genetic risk score level.

Abbreviations: Adipo-IR, adipose tissue insulin resistance; AUC, area under the curve; DM, diabetes mellitus; FFA, free fatty acids; HDL-C, high-density lipoprotein cholesterol; HOMA-IR, homeostatic model assessment of insulin resistance; LDL-C, low-density lipoprotein cholesterol.

^*a*^The Jonckheere-Terpstra test was performed in the non-DM and DM groups to calculate *P* trends.

^*b*^ *P* less than .05 vs non-DM at the same genetic risk score level.

**Table 5.** Comparison of biochemical characteristics in nondiabetes mellitus and diabetes mellitus groups by genetic risk score level in female participants (n = 931)

	Low risk		Average risk		High risk		*P* trend*^a^*	
	Non-DM	DM	Non-DM	DM	Non-DM	DM	Non-DM	DM
n = 297		n = 13	n = 278	n = 32	n = 271	n = 40		
Glucose, mg/dL	92.0 (87.0-98.0)	120.0 (108.5-134.5)*^b^*	94.0 (89.0-99.0)	127.0 (105.5-152.0)*^b^*	94.0 (90.0-101.0)	130.5 (120.8-146.8)*^b^*	<.001	.116
Glucose-AUC, mg/dL × 10^−4^	1.7 (1.4-2.0)	2.8 (2.3-3.0)*^b^*	1.7 (1.5-2.0)	2.9 (2.6-3.2)*^b^*	1.8 (1.6-2.1)	2.9 (2.5-3.4)*^b^*	<.001	.701
HbA_1c_, %	5.6 (5.5-5.8)	6.6 (6.4-7.3)*^b^*	5.7 (5.5-5.9)	6.8 (6.5-7.5)*^b^*	5.7 (5.5-6.0)	7.0 (6.5-7.4)*^b^*	<.001	.256
Fasting insulin, μU/mL	4.1 (2.8-6.1)	5.4 (3.8-6.9)	4.0 (2.6-5.4)	4.8 (3.3-6.8)*^b^*	3.9 (2.8-5.8)	4.7 (2.9-7.3)	.645	.475
Insulin-AUC, μU/mL × 10^−3^	5.0 (3.9-7.0)	3.1 (2.5-4.8)*^b^*	5.1 (3.7-7.0)	3.0 (2.3-4.2)*^b^*	4.9 (3.6-7.0)	3.5 (2.5-5.6)*^b^*	.534	.364
HOMA-IR	0.9 (0.6-1.4)	1.4 (1.1-2.0)*^b^*	0.9 (0.6-1.3)	1.5 (1.0-2.3)*^b^*	0.9 (0.6-1.4)	1.4 (0.8-2.5)*^b^*	.926	.881
Matsuda index	6.9 (4.8-9.6)	5.2 (3.7-6.7)	6.7 (4.8-9.8)	5.6 (4.1-6.9)*^b^*	6.8 (4.6-9.2)	4.8 (3.0-7.8)*^b^*	.648	.615
Insulinogenic index	0.7 (0.4-1.1)	0.1 (0.1-0.2)*^b^*	0.6 (0.4-1.0)	0.2 (0.1-0.3)*^b^*	0.5 (0.3-0.9)	0.2 (0.1-0.3)*^b^*	<.001	.264
C-peptide, ng/mL	1.2 (1.0-1.6)	1.5 (1.4-1.8)*^b^*	1.2 (0.9-1.5)	1.4 (1.2-2.0)*^b^*	1.2 (1.0-1.6)	1.6 (1.0-2.0)*^b^*	.581	.993
AUC-C-peptide, ng/mL × 10^−2^	7.4 (6.3-9.1)	6.1 (4.9-7.4)*^b^*	7.6 (6.2-9.2)	5.2 (4.6-6.6)*^b^*	7.5 (6.2-9.1)	6.3 (4.7-7.6)*^b^*	.958	.360
Fasting FFA, µmol/L	492.0 (385.1-629.0)	501.7 (370.0-668.0)	497.3 (377.0-642.4)	628.1 (548.2-807.6)*^b^*	528.0 (394.0-668.5)	540.3 (446.9-673.9)	.157	.584
FFA-AUC, µmol/L × 10^−4^	2.7 (2.1-3.5)	4.1 (3.1-5.0)*^b^*	2.8 (2.1-3.4)	4.8 (3.8-5.8)*^b^*	3.1 (2.3-3.7)	4.2 (3.5-5.3)*^b^*	.019	.615
Adipo-IR	2.0 (1.2-3.4)	3.0 (1.2-4.5)	1.9 (1.1-3.1)	2.9 (2.1-4.9)*^b^*	2.0 (1.3-3.5)	2.3 (1.3-4.5)	.688	.475
Total cholesterol, mg/dL	213.0 (193.0-238.0)	224.0 (189.5-235.5)	214.0 (193.0-238.0)	202.0 (181.0-216.0)*^b^*	215.0 (193.0-238.0)	207.0 (181.3-227.8)*^b^*	.730	.442
HDL-C, mg/dL	66.0 (57.0-77.0)	60.0 (55.0-72.0)	69.0 (59.0-80.0)	63.5 (49.3-76.0)	67.0 (57.0-78.0)	58.0 (49.3-69.8)*^b^*	.553	.262
LDL-C, mg/dL	128.0 (109.0-149.0)	138.0 (108.0-154.0)	124.0 (105.8-147.0)	118.5 (99.3-132.5)	125.0 (106.0-147.5)	113.5 (95.8-134.3)*^b^*	.527	.211
Triglycerides, mg/dL	81.0 (63.0-109.5)	89.0 (68.5-144.5)	79.0 (59.8-107.0)	94.5 (80.8-128.0)*^b^*	80.0 (60.0-113.0)	108.0 (81.8-150.5)*^b^*	.953	.181

Data are presented as median (quartiles); the *t* test and Mann-Whitney *U* test were performed to compare non-DM and DM groups within the same genetic risk score level.

Abbreviations: Adipo-IR, adipose tissue insulin resistance; AUC, area under the curve; DM, diabetes mellitus; FFA, free fatty acids; HDL-C, high-density lipoprotein cholesterol; HOMA-IR, homeostatic model assessment of insulin resistance; LDL-C, low-density lipoprotein cholesterol.

^*a*^The Jonckheere-Terpstra test was performed in the Non-DM and DM groups to calculate *P* trends.

^*b*^ *P* less than .05 vs non-DM at the same genetic risk score level.

### Trend of Characteristics Within Nondiabetes Mellitus and Diabetes Mellitus Groups Through 3 Levels of Genetic Risk Score

The results of the Jonckheere-Terpstra test confirmed a trend of decreasing VFA with increasing GRS in men in the DM group, but not in women (see Table 3). In men, higher GRS were also associated with lower CPR values both in the non-DM and DM groups. Also, there was a decreasing trend in the II and triglyceride values in the non-DM group. In women, higher GRS was associated with lower II values and higher FFA-AUC values in the non-DM group (see Table 4). On the other hand, higher GRS were not associated with any changes in HOMA-IR and Matsuda index values in either the non-DM or DM group.

## Discussion

This study incorporated 110 SNVs into the GRS for Japanese T2D and showed that higher GRS levels were associated with a higher prevalence of T2D even after adjustment for confounding factors such as age, BMI, body composition, and physical activity levels. These data confirmed the GRS used in this study is associated with the prevalence of T2D. Compared with the non-DM group, the DM group exhibited higher VFA and HOMA-IR values and lower II values across all genetic risk levels. In the non-DM group, the II value decreased with increasing GRS levels. Meanwhile, the transition of GRS was not associated with any substantial change in the values of the HOMA-IR or Matsuda index, both of which are indicators of insulin resistance. These data suggest that the GRS mainly predicts a decrease in β-cell function.

T2D is considered to develop long before its clinical signs become apparent [15], underscoring the clinical value of its early detection and highlighting the importance of identifying high-risk individuals. Furthermore, since the GRS can assess risk at the level of each individual, it represents a substantial advancement in early-risk prediction. Following the discovery of an increasing number of SNVs, previous studies have explored the relationship between the GRS and the risk of T2D development, revealing improvements in predictive ability across various ethnic populations [16-18]. Similarly, in the Japanese population, the utility of the GRS has been examined whenever new SNVs have been discovered [6, 19, 20]. Building on this evidence, our study used loci derived from a large-scale GWAS of Japanese individuals (n∼200 000), potentially leading to higher accuracy [5]. While our analysis did not directly assess the predictive ability of the GRS in this cohort, our findings underscore a notably strong association between the GRS and the risk of T2D in older Japanese adults, even after a thorough adjustment for several potential confounders (age, BMI, PBF, VFA, and physical activity levels). This outcome highlights the robustness of our data set in providing valuable insights into the genetic determinants of T2D susceptibility in the Japanese population. Furthermore, the ORs for T2D prevalence remained largely unchanged even after adjusting for potential confounders. This may be attributed to the similarity in sociodemographic characteristics of the study participants across the 3 GRS levels, except for the prevalence of hypertension. This finding suggests that the SNVs included in this study were specifically associated with the T2D trait and did not significantly affect other demographic characteristics such as median age, height, BMI, and other traits. Previous studies on GRS for T2D in Japan have demonstrated similar results [19]. Moreover, this emphasizes the notion that individuals may exhibit varying genetic risks despite sharing similar sociodemographic characteristics.

On comparing the DM and non-DM groups at each genetic risk level, we observed that older adults with diabetes in our study exhibited a lower insulinogenic index, higher VFA, and higher indices of insulin resistance compared with those without diabetes, findings that were consistent across all GRS levels. VFA, which is linked to insulin resistance, contributes considerably to the development of T2DM [21, 22]. Previous studies on the GRS have consistently demonstrated that it is strongly associated with decreased insulin secretion, but that it generally shows a weaker or negligible relationship with insulin resistance [23-25]. Therefore, we propose the presence of a cumulative risk effect, whereby our participants' progression to T2D may primarily stem from decreased insulin secretion as the GRS increases, compounded by the exacerbating factor of higher VFA contributing to increased insulin resistance. This was confirmed by decreasing VFA with increasing GRS in male participants with diabetes, suggesting that individuals with a high GRS may develop T2D at a lower VFA than those with a low GRS. This dual pathway—genetic predisposition decreasing insulin secretion, and metabolic dysfunction increasing insulin resistance—underscores the importance of individualized approaches to preventing T2D. However, further research is required to confirm our hypothesis.

In addition to the insulin resistance index and VFA mentioned earlier, the DM group had lower HDL-C, higher triglyceride levels, and higher FFA-AUC values compared with the non-DM group. These findings are considered to result from lipid spill-over associated with the accumulation of VFA due to reduced physical activity and overeating [26-28]. Further, previous studies showed that lifestyle modification can have a positive effect on reducing VFA and triglycerides, as well as increasing insulin sensitivity and HDL-C [29-33]. However, in this study, total energy intake and physical activity level were comparable between the DM and non-DM groups. Based on the proven connection between a positive lifestyle and improvement in the aforementioned markers, we anticipate that in the present study, there was bias in participants' responses to the diet history and physical activity questionnaire; specifically, participants with diabetes may have underreported their dietary consumption or overestimated their physical activity level [34, 35]. On the other hand, irrespective of an individual's genetic risk of diabetes, interventions through weight loss, physical activity, and dietary restriction have been proven to be beneficial in preventing the onset of diabetes [36], and it is advisable to continue using these markers.

### Limitations

There are several limitations to this study. First, this was a cross-sectional study, and therefore, while we adjusted for some confounding factors when estimating ORs, not all potential confounding can be eliminated. Second, given that the recruited individuals were able to visit our research facility, there was inevitable selection bias that affected the health representativeness of this study's participants for the Japanese older adults. Notably, however, we confirmed that the participants had the same age distribution as the older adult populations in Bunkyo-ku and Tokyo. Third, we excluded individuals with T2D who were using insulin, as insulin therapy could influence blood test results and potentially introduce confounding variables. However, this exclusion may limit the generalizability of our findings to the broader T2D population, especially those with advanced disease who are more likely to require insulin. Fourth, given the multiple comparisons conducted in this study, there is a potential risk of type I error. Therefore, the statistically significant findings should be interpreted with caution, and further investigation, including future studies, is needed to validate these results. Finally, there may have been recall bias concerning physical activity and dietary intake, since both were assessed using a questionnaire.

### Conclusion

Our study demonstrated that a higher GRS was significantly associated with an increased prevalence of T2D in older Japanese individuals, independently of confounding factors. The findings suggest that a higher GRS is linked to lower insulin secretion rather than increased insulin resistance. Given that insulin resistance and increased VFA are reversible factors, they may be critical targets for preventing T2D, particularly in older adults at high genetic risk. This emphasizes the importance of early detection and individualized prevention strategies to mitigate the risk of T2D.

## Data Availability

Some or all data sets generated during and/or analyzed during the present study are not publicly available but are available from the corresponding author on reasonable request.

## Abbreviations

Adipo-IR: adipose tissue insulin resistance
AUC: area under the curve
BHS: Bunkyo Health Study
BMI: body mass index
CPR: C-peptide immunoreactivity
DM: diabetes mellitus
FFA: free fatty acids
FPG: fasting plasma glucose
GRS: genetic risk scores
GWASs: genome-wide association studies
HbA_1c_: glycated hemoglobin A_1c_
HDL-C: high-density lipoprotein cholesterol
HOMA-IR: homeostatic model assessment of insulin resistance
II: insulinogenic index
LDL-C: low-density lipoprotein cholesterol
METs: metabolic equivalents
OGTT: oral glucose tolerance test
OR: odds ratio
PBF: percentage of body fat
SNV: single-nucleotide variations
T2D: type 2 diabetes
VFA: visceral fat area
